# Estimation of breeding values for uniformity of growth in Atlantic salmon (*Salmo salar*) using pedigree relationships or single-step genomic evaluation

**DOI:** 10.1186/s12711-017-0308-3

**Published:** 2017-03-07

**Authors:** Panya Sae-Lim, Antti Kause, Marie Lillehammer, Han A. Mulder

**Affiliations:** 1 Nofima Ås, Osloveien 1, P.O. Box 210, 1431 Ås, Norway; 2 Biometrical Genetics, Natural Resources Institute Finland, 31600 Jokioinen, Finland; 3 Animal Breeding and Genomics Centre, Wageningen University and Research, P.O. Box 338, 6700 AH Wageningen, The Netherlands

## Abstract

**Background:**

In farmed Atlantic salmon, heritability for uniformity of body weight is low, indicating that the accuracy of estimated breeding values (EBV) may be low. The use of genomic information could be one way to increase accuracy and, hence, obtain greater response to selection. Genomic information can be merged with pedigree information to construct a combined relationship matrix (${\mathbf{H}}$ matrix) for a single-step genomic evaluation (ssGBLUP), allowing realized relationships of the genotyped animals to be exploited, in addition to numerator pedigree relationships (${\mathbf{A}}$ matrix). We compared the predictive ability of EBV for uniformity of body weight in Atlantic salmon, when implementing either the ${\mathbf{A}}$ or ${\mathbf{H}}$ matrix in the genetic evaluation. We used double hierarchical generalized linear models (DHGLM) based either on a sire-dam (sire-dam DHGLM) or an animal model (animal DHGLM) for both body weight and its uniformity.

**Results:**

With the animal DHGLM, the use of ${\mathbf{H}}$ instead of ${\mathbf{A}}$ significantly increased the correlation between the predicted EBV and adjusted phenotypes, which is a measure of predictive ability, for both body weight and its uniformity (41.1 to 78.1%). When log-transformed body weights were used to account for a scale effect, the use of ${\mathbf{H}}$ instead of ${\mathbf{A}}$ produced a small and non-significant increase (1.3 to 13.9%) in predictive ability. The sire-dam DHGLM had lower predictive ability for uniformity compared to the animal DHGLM.

**Conclusions:**

Use of the combined numerator and genomic relationship matrix (${\mathbf{H}}$) significantly increased the predictive ability of EBV for uniformity when using the animal DHGLM for untransformed body weight. The increase was only minor when using log-transformed body weights, which may be due to the lower heritability of scaled uniformity, the lower genetic correlation of transformed body weight with its uniformity compared to the untransformed traits, and the small number of genotyped animals in the reference population. This study shows that ssGBLUP increases the accuracy of EBV for uniformity of body weight and is expected to increase response to selection in uniformity.

**Electronic supplementary material:**

The online version of this article (doi:10.1186/s12711-017-0308-3) contains supplementary material, which is available to authorized users.

## Background

In aquaculture, selection to increase economically important traits such as growth is one of the main breeding goals. However, fish producers show interest to improve not only the mean but also the variance of traits [1]. Uniformity of growth is preferable because more uniform growth allows a more uniform product, harvest of a larger proportion of the population at market size, and reduction of size grading and multiple harvests [2–4]. More uniform growth may also reduce competitive interactions between animals, which contributes to reduce feed monopolization and dominant behaviour, and thus improve well-being of fish [5]. Uniformity is also important for traits that have an intermediate optimal trait value [6], such as fillet lipid%, body shape, and condition factor in the aquaculture industry. A fish whose growth is sensitive to non-measurable environmental factors, known as micro-environments, shows micro-environmental sensitivity, which results in high environmental variance and consequently contributes to increased phenotypic variation, leading to increased size variation within a group of fish. A number of empirical studies in terrestrial and aquatic species show that uniformity is partly determined by genetic factors [4, 7–16]. Thus, selective breeding can open up one avenue to improve uniformity of fish traits.

Atlantic salmon (*Salmo salar* L.) is a farmed fish that is of major economic importance. Heritability for uniformity of body weight has been estimated in Atlantic salmon [14], rainbow trout (*Oncorhynchus mykiss* Walbaum) [4, 8], and Nile tilapia (*Oreochromis niloticus*) [15, 16]. In general, heritability for uniformity ($h_{v}^{2}$) is low in livestock and aquaculture species ($h_{v}^{2}$ < 0.05), indicating that the prediction accuracy of breeding values for uniformity may be low [17, 18]. However, the coefficient of genetic variation ($GCV$) of uniformity of body weight is high in fish species (median $GCV$ = 34.0%: min = 17.4% and max = 64.0%), which indicates high potential for response to selection [4, 8, 14, 16, 19]. One way to increase response to selection for uniformity is to increase the accuracy of estimated breeding values (EBV) for uniformity [20].

In aquaculture, full- and half-sib family sizes are usually large and thus the accuracy of EBV based on full-sibs, half-sibs and own performance is high for body weight, but not for uniformity due to its low heritability [8]. One approach to increase the accuracy of EBV is to use genomic information [21]. With genomic selection, genomic estimated breeding values (GEBV) can be obtained for the selection candidates that are genotyped, even when they have no phenotype records. One reason why genomic selection results in higher accuracy of selection is the more accurate estimation of the Mendelian sampling genetic effects through realized additive genetic relationships among animals [22]. Consequently, individual squared residuals, which is the phenotype for uniformity in a double hierarchical generalized linear model (DHGLM), may also be more accurately estimated when using genomic information.

In many cases, combining numerator pedigree and genomic information in genomic evaluations is implemented in multiple steps, which may introduce bias and need some calculations to combine with pedigree-based EBV [23, 24]. Single step genomic best linear unbiased prediction (ssGBLUP) avoids this, and genomic and pedigree information are combined in one step [23], which may lead to less bias and is less prone to double counting of information compared to genomic evaluation methods that are performed in multiple steps. The ssGBLUP augments the numerator relationship (${\mathbf{A}}$) matrix by the genomic relationship (${\mathbf{G}}$) matrix in conventional genetic evaluation using BLUP [24]. This combined numerator and genomic relationship matrix is known as the ${\mathbf{H}}$ matrix [25]. In fish breeding, combining pedigree and genomic information allows exploiting the large full- and half-sib families and the more accurate relationships of the genotyped animals, and may yield a higher accuracy of selection for uniformity than the use of the ${\mathbf{A}}$ matrix.

To date, the use of ssGBLUP for uniformity has not been studied. Furthermore, according to a previous study, the sire-dam model, but not the animal model, implemented within the framework of DHGLM provided unbiased (co)variance component estimates [14]. However, an animal DHGLM is expected to perform better than a sire-dam DHGLM for genetic evaluation because the animal DHGLM uses full relationships between animals rather than only among sires and dams. This is particularly important for uniformity, which is quantified by the residuals of individuals, which in the animal model do not contain the Mendelian sampling term. Moreover, for genetic evaluation, the animal DHGLM uses all phenotypic information and, for most breeding programs, at least part of the selection candidates, e.g. females for sex-linked traits, have phenotypes available at the time of selection. Use of the animal DHGLM with ssGBLUP for uniformity has not been tested.

In this study, we implemented ssGBLUP for predicting GEBV for uniformity in Atlantic salmon. Specifically, our aim was to compare the predictive ability of EBV for uniformity of body weight when implementing either BLUP with the ${\mathbf{A}}$ matrix or ssGBLUP with the ${\mathbf{H}}$ matrix. The (co)variance components were estimated from the sire-dam DHGLM with either ${\mathbf{A}}$ or ${\mathbf{H}}$ and compared prior to genetic evaluation.

## Methods

### Data

The data used in this study originated from the experiment conducted by Nofima AS and the breeding company SalmoBreed in Norway. The experiment followed all the regulations of animal ethical practice and was approved by the Norwegian Research Animal Committee (ID 6489). In 2013, 234 full-sib families were established from the mating of 131 sires to 234 dams (Table 1) during four weeks. Forty-seven percent of the parents were from year class 2009 and the rest from year class 2010. After hatching, fingerlings from each family were held in a 180-L family tank until tagging size (at mean body weight of 50 g). Each animal was tagged using passive integrated transponder (PIT) tags (Satpos AS, Norway). During tagging, a fin sample for genotyping was collected from 21 to 38 sibs of each of 50 full-sib families. Thereafter, all fish were randomly allocated to three experiment tanks and grown for 11 months. At the average age of 16 months, all fish were challenged with sea lice using a co-habitat challenge, and at the end of the challenge test, final body weight (g) was measured for all 3595 fish with an electronic balance. A total of 1416 offspring (39% of all offspring) and the 131 sires and 234 dams were genotyped using the 31 K Affymetrix single nucleotide polymorphism (SNP) chip for Atlantic salmon developed by Nofima. Quality control of SNPs was performed in PLINK v1.9 [26] based on the following criteria: SNPs were removed if (1) their call rate was lower than 90%, (2) they deviated from Hardy–Weinberg equilibrium with a *P* value cut-off of 10^−15^, and (3) their minor allele frequency (MAF) was lower than 0.01. After quality control, 921 of 31,013 SNPs were removed (2.9%) and, thus 30,092 SNPs remained to create the genomic relationship.

**Table 1:** Population structure of Atlantic salmon

Population structure	
Sires, dams	131, 234
Sires per dam, mean (range)	1.0 (1.0)
Dams per sire, mean (range)	1.78 (1–3)
Full-sib families	234
Fish per full-sib family, mean (range)	15.4 (4–54)
Number of fish with records	3595

Genotyped animals	
Full-sib families	50
Fish per full-sib family, mean (range)	28.3 (21–38)
Number of fish with records	1416

### Relationship matrix

The numerator relationship (${\mathbf{A}}$) matrix with 814 ancestors in four generations was prepared based on pedigree information using ASReml [27]. The combined numerator and genomic relationship (${\mathbf{H}}$) matrix was defined as [23]:

$${\mathbf{H}} = \left[ {\begin{array}{*{20}c} {{\mathbf{A}}_{11} + {\mathbf{A}}_{12} + {\mathbf{A}}_{22}^{ - 1} \left( {{\mathbf{G}} - {\mathbf{A}}_{22} } \right){\mathbf{A}}_{22}^{ - 1} {\mathbf{A}}_{21} } & {{\mathbf{A}}_{12} {\mathbf{A}}_{22}^{ - 1} {\mathbf{G}}} \\ {{\mathbf{GA}}_{22}^{ - 1} {\mathbf{A}}_{21} } & {\mathbf{G}} \\ \end{array} } \right],$$

where ${\mathbf{A}}_{11}$ is the pedigree relationship matrix between non-genotyped animals, ${\mathbf{A}}_{12}$ and ${\mathbf{A}}_{21}$ are pedigree relationship matrices between genotyped and non-genotyped animals, ${\mathbf{A}}_{22}$ is the pedigree relationship matrix between genotyped animals, and ${\mathbf{G}}$ is the genomic relationship matrix between genotyped animals. The ${\mathbf{G}}$ matrix was computed as [28]: ${\mathbf{G}} = \frac{{{\mathbf{WW^{\prime}}}}}{N}$, where ${\mathbf{W}}$ is the matrix of the scaled SNP genotypes for all loci and $N$ is the total number of SNPs (30,092). The elements of ${\mathbf{W}}$ were calculated as:

$$w_{ij} = \frac{{\left( {x_{ij} - 2p_{j} } \right)}}{{\sqrt {2p_{j} \left( {1 - p_{j} } \right)} }},$$

where $x_{ij}$ is the SNP genotype (coded 0, 1, or 2) for the $i$th individual at SNP $j$ and $p_{j}$ is the allele frequency of the homozygous genotype coded as 2.

However, Aguilar et al. [29] and Christensen and Lund [30] showed that the inverse of the ${\mathbf{H}}$ matrix can be computed as:

$${\mathbf{H}}^{ - 1} = {\mathbf{A}}^{ - 1} + \left[ {\begin{array}{*{20}c} {\mathbf{0}} & {\mathbf{0}} \\ {\mathbf{0}} & {{\mathbf{G}}^{ - 1} - {\mathbf{A}}_{22}^{ - 1} } \\ \end{array} } \right],$$

which is less computational demanding and more simple than preparing and subsequently inverting the ${\mathbf{H}}$ matrix. The ${\mathbf{H}}^{ - 1}$ was prepared by using the Calc_grm computer software [31], which prepares both ${\mathbf{A}}^{ - 1}$ and ${\mathbf{G}}^{ - 1}$ internally before computing ${\mathbf{H}}^{ - 1}$.

### Statistical analysis

#### Analysis of residuals

Uniformity can be quantified by squared residuals from a BLUP mixed model equation [32]. The use of genomic information to construct realised relationships between animals, especially for full-sibs, is expected to increase the accuracy of residual estimates due to a greater accuracy of EBV for body weight. Therefore, we investigated the effect of ssGBLUP and traditional BLUP on individual residual estimation. Furthermore, we investigated sire-dam and animal models because residual estimates from a sire-dam model contain not only the unexplained environmental effects but also Mendelian sampling genetic effects. Residual estimates from an animal model do not contain the latter when EBV are estimated with an accuracy of 1. In total, residuals from four models were compared, i.e. the sire-dam or animal model with either ${\mathbf{A}}$ or ${\mathbf{H}}$.

The animal mixed model was:

1 $$y_{iklmn } = \mu + \beta age_{k} + t_{l} + yc_{m} + a_{i} + c_{n} + e_{iklmn} ,$$

where $y_{iklmn }$ is the observation (body weight) of the *i*th individual, $\mu$ is the overall mean, $age$ is the fixed covariate effect due to different levels of age of the fish, calculated from the start feeding date until the date of measurement (day), $\beta$ is the fixed linear regression coefficient on age, $t$ is the $l$th fixed communal tank effect, $yc$ is the $m$th fixed effect of year class of the parents, $a_{i}$ is the random additive genetic effect, $\varvec{a}\sim\,\left[ {0, {\mathbf{A}}\sigma_{a}^{2} } \right]$, where ${\mathbf{A}}$ is the numerator relationship matrix, or $\varvec{a}\sim\,N\left[ {0, {\mathbf{H}}\sigma_{a}^{2} } \right]$, where ${\mathbf{H}}$ is the combined genomic and pedigree relationship matrix, $N$ is the normal distribution, and $\sigma_{a}^{2}$ is the additive genetic variance for body weight, $c_{n}$ is the random common effect for full-sibs, $\varvec{c}\sim{\text{N}}\left[ {0, {\mathbf{I}}\sigma_{c}^{2} } \right],$ where ${\mathbf{I}}$ is the identity matrix and $\sigma_{c}^{2}$ is the common environmental variance of body weight, and $e_{iklmn}$ is the random residual effect, $\varvec{e}\sim\,N\left[ {0, {\mathbf{I}}\sigma_{e}^{2} } \right]$, where $\sigma_{e}^{2}$ is the residual variance of body weight assumed to be homogeneous. For the sire-dam model, the term $a_{i}$ in Eq. (1) was replaced by the random sire-dam ($u_{i}$) effect, $\varvec{u}\sim\,N\left[ {0, {\mathbf{A}}\sigma_{u}^{2} } \right]$ or $\varvec{u}\sim\,N\left[ {0,{\mathbf{H}}\sigma_{u}^{2} } \right]$. The same ${\mathbf{A}}$ and ${\mathbf{H}}$ matrices were used for the sire-dam and the animal models.

#### Estimation of genetic parameters for uniformity

To estimate genetic parameters for body weight and its uniformity, the sire-dam DHGLM was used [33, 34] because it is expected to provide unbiased (co)variance components for uniformity [14]. Body weight records were treated in two different ways. First, observed body weight was standardized to a mean of 0 and variance of 1, which facilitates convergence of the DHGLM. Second, we used either the natural log or the Box–Cox transformation to account for possible scale effects, because variances typically increase with increasing trait means [35, 36]. For the Box–Cox transformation, each observation was computed as $\frac{{y_{i}^{\lambda } - 1}}{\lambda }$, where $\lambda$ is the transformation parameter, which was estimated based on Eq. (1) without the random effects [37] by maximum likelihood using the MASS package in R software [38]. The estimate of $\lambda$ was close to 0 (0.076), indicating that the Box–Cox transformation is very similar to log-transformation, which sets $\lambda$ equal to 0. Therefore, the Box–Cox transformed body weight was not used further. The standardized body weight and natural logarithm body weight are abbreviated as stdWT and lnWT, respectively.

To estimate genetic parameters, standardized and transformed body weights were modelled using sire-dam DHGLM in ASReml [32]:

2 $$\begin{aligned} \left[ {\begin{array}{*{20}c} {\mathbf{y}} \\ {\varvec{\Psi}} \\ \end{array} } \right] & = \left[ {\begin{array}{*{20}c} {\mathbf{X}} & {\mathbf{0}} \\ {\mathbf{0}} & {{\mathbf{X}}_{v} } \\ \end{array} } \right]\left[ {\begin{array}{*{20}c} {\mathbf{b}} \\ { {\mathbf{b}}_{v} } \\ \end{array} } \right] + \left[ {\begin{array}{*{20}c} {\left( {{\mathbf{Z}}_{\varvec{s}} + {\mathbf{Z}}_{\varvec{d}} } \right)} & {\mathbf{0}} \\ {\mathbf{0}} & {\left( {{\mathbf{Z}}_{\varvec{s}} + {\mathbf{Z}}_{\varvec{d}} } \right)_{v} } \\ \end{array} } \right] \\ & \quad \times \left[ {\begin{array}{*{20}c} {\mathbf{u}} \\ {\varvec{ }{\mathbf{u}}_{v} } \\ \end{array} } \right] + \left[ {\begin{array}{*{20}c} {\mathbf{Q}} & {\mathbf{0}} \\ {\mathbf{0}} & {{\mathbf{Q}}_{v} } \\ \end{array} } \right]\left[ {\begin{array}{*{20}c} {\mathbf{c}} \\ { {\mathbf{c}}_{v} } \\ \end{array} } \right] + \left[ {\begin{array}{*{20}c} {\mathbf{e}} \\ { {\mathbf{e}}_{v} } \\ \end{array} } \right], \\ \end{aligned}$$

where ${\mathbf{y}}$ is the vector of stdWT or lnWT records for the $i$th individual; ${\varvec{\Psi}}$ is the vector of response variables for the residual variance, where $\psi_{i} = \log \left( {\hat{\sigma }_{{e_{i} }}^{2} } \right) + \frac{{\frac{{\hat{e}_{i}^{2} }}{{1 - h_{i} }} - \hat{\sigma }_{{e_{i} }}^{2} }}{{\hat{\sigma }_{{e_{i} }}^{2} }}$, which was linearized using a Taylor series approximation in ASReml [34], $\hat{e}_{i}^{2}$ is the squared residual of the $i$th body weight record, $h_{i}$ is the diagonal element in the hat-matrix of ${\mathbf{y}}$ [39], and $\hat{\sigma }_{{e_{i} }}^{2}$ is the estimated residual variance of the $i$th observation in the previous iteration of ASReml; ${\mathbf{X}}$ and ${\mathbf{X}}_{v}$ are incidence matrices of the fixed effects described in Eq. (1) for the trait mean and its uniformity, respectively; ${\mathbf{b}}$ (${\mathbf{b}}_{v}$) is the solution vector for the corresponding fixed effects; ${\mathbf{Z}}_{\varvec{s}}$ and ${\mathbf{Z}}_{\varvec{d}}$ are incidence matrices for the random sire (s) and dam (d) effects; ${\mathbf{u}}$ (${\mathbf{u}}_{v}$) is the vector of additive genetic effects of sire-dam on the weight (uniformity), which was assumed to follow a normal distribution for the ${\mathbf{A}}$ matrix:

$$\left[ {\begin{array}{*{20}c} {\mathbf{u}} \\ {\varvec{ }{\mathbf{u}}_{v} } \\ \end{array} } \right]\sim{\text{N}}\left( {\left[ {\begin{array}{*{20}c} {\mathbf{0}} \\ {\mathbf{0}} \\ \end{array} } \right], \frac{1}{4}\left[ {\begin{array}{*{20}c} {\sigma_{a}^{2} } & {\sigma_{{a,a_{v} ,exp}} } \\ {\sigma_{{a,a_{v} ,exp}} } & {\sigma_{{a_{v} ,exp}}^{2} } \\ \end{array} } \right] \otimes {\mathbf{A}}} \right),$$

and for the ${\mathbf{H}}$ matrix:

$$\left[ {\begin{array}{*{20}c} {\mathbf{u}} \\ {\varvec{ }{\mathbf{u}}_{v} } \\ \end{array} } \right]\sim{\text{N}}\left( {\left[ {\begin{array}{*{20}c} {\mathbf{0}} \\ {\mathbf{0}} \\ \end{array} } \right], \frac{1}{4}\left[ {\begin{array}{*{20}c} {\sigma_{a}^{2} } & {\sigma_{{a,a_{v} ,exp}} } \\ {\sigma_{{a,a_{v} ,exp}} } & {\sigma_{{a_{v} ,exp}}^{2} } \\ \end{array} } \right] \otimes {\mathbf{H}}} \right),$$

where the $\frac{1}{4}$ accounts for the fact that the sire and dam each explain only a quarter of the additive genetic variance; ${\mathbf{Q}}$ (${\mathbf{Q}}_{v}$) is the incidence matrix for the random common effects to full-sibs; ${\mathbf{c}}$ (${\mathbf{c}}_{v}$) is the vector of common effects to full-sibs:

$$\left[ {\begin{array}{*{20}c} {\mathbf{c}} \\ {\varvec{ }{\mathbf{c}}_{v} } \\ \end{array} } \right]\sim{\text{N}}\left[ {\left[ {\begin{array}{*{20}c} {\mathbf{0}} \\ {\mathbf{0}} \\ \end{array} } \right], \left[ {\begin{array}{*{20}c} {\sigma_{c}^{2} } & {\sigma_{{c,c_{v} ,exp}} } \\ {\sigma_{{c,c_{v} ,exp}} } & {\sigma_{{c_{v} ,exp}}^{2} } \\ \end{array} } \right] \otimes {\mathbf{I}}} \right].$$

The residuals of ${\mathbf{y}}$ (${\mathbf{e}}$) and ${\varvec{\Psi}}$ (${\mathbf{e}}_{v}$) were assumed to be independently normally distributed as follows:

$$\left[ {\begin{array}{*{20}c} {\mathbf{e}} \\ { {\mathbf{e}}_{v} } \\ \end{array} } \right]\sim{\text{N}}\left( {\left[ {\begin{array}{*{20}c} {\mathbf{0}} \\ {\mathbf{0}} \\ \end{array} } \right],\left[ {\begin{array}{*{20}c} {{\mathbf{W}}^{ - 1} \sigma_{\epsilon}^{2} } & {\mathbf{0}} \\ {\mathbf{0}} & {{\mathbf{W}}_{v}^{ - 1} \sigma_{{\epsilon\ominus_{v} }}^{2} } \\ \end{array} } \right]} \right),$$

where ${\mathbf{W}} = {\text{diag}}({\hat{{\varvec{\Psi}}}}^{ - 1} )$ and ${\mathbf{W}}_{v} = {\text{diag}}\left( {\frac{{1 - {\mathbf{h}}}}{2}} \right)$, and $\sigma_{\epsilon}^{2}$ ($\sigma_{{\epsilon_{v} }}^{2}$) is a scaled variance that was expected to be 1. The sire-dam DHGLM was fitted iteratively to update ${\varvec{\Psi}}$, ${\text{diag}}\left( {\mathbf{W}} \right)$ and ${\text{diag}}\left( {{\mathbf{W}}_{v} } \right)$ until the log-likelihood converged [34].

### Calculation of genetic parameters

In the sire-dam DHGLM, the estimated variance for sires was set equal to the estimated genetic variance for dams and equal to one quarter of the additive genetic variance. Hence, the additive genetic variance for body weight ($\sigma_{a}^{2}$) and its uniformity ($\sigma_{{a_{v} ,exp}}^{2}$) were equal to $4\sigma_{u}^{2}$ and $4\sigma_{{u_{v} ,exp}}^{2}$, respectively. Estimates for $\sigma_{{u_{v} ,exp}}^{2}$ and $\sigma_{{c_{v} ,exp}}^{2}$ for uniformity of body weight were on the exponential scale ($exp$) and were converted to an additive scale ($\sigma_{{u_{v} }}^{2}$ and $\sigma_{{c_{v} }}^{2}$) using the extension of the equations of Mulder et al. [17], as derived by Sae-Lim et al. [8]. The additive genetic variance for uniformity of body weight on the additive scale was equal to $4\sigma_{{u_{v} }}^{2}$. Phenotypic variance ($\sigma_{P}^{2}$) of body weight was equal to $2\sigma_{u}^{2} + \sigma_{c}^{2} + \sigma_{e}^{2}$, where $\sigma_{c}^{2}$ is the variance component for the effect common to full-sibs and $\sigma_{e}^{2}$ is the residual variance of body weight. Heritability for body weight ($h^{2}$) was calculated as $\sigma_{a}^{2} /\sigma_{P}^{2}$. Heritability for uniformity of body weight ($h_{v}^{2}$) on the additive scale was calculated as $\frac{{\sigma_{{a_{v} }}^{2} }}{{2\sigma_{P}^{4} + 3\left( {\sigma_{{a_{v} }}^{2} + \sigma_{{c_{v} }}^{2} } \right)}}$ [8, 40]. Similarly, the common environmental effect was calculated as $c^{2} = \sigma_{c}^{2} /\sigma_{P}^{2}$ for body weight and as $c_{v}^{2} =$ $\frac{{\sigma_{{c_{v} }}^{2} }}{{2\sigma_{P}^{4} + 3\left( {\sigma_{{a_{v} }}^{2} + \sigma_{{c_{v} }}^{2} } \right)}}$ for uniformity of body weight [8]. The genetic coefficient of variation for uniformity of body weight ($GCV$) was calculated as $\sqrt {\sigma_{{a_{v} ,exp}}^{2} }$. Standard errors of $h_{v}^{2}$ and $GCV$ were approximated using the equations presented by Mulder et al. [41].

### Genetic evaluation and cross-validation

Two genetic evaluations, i.e., BLUP with ${\mathbf{A}}$ and ssGBLUP with ${\mathbf{H}}$, were performed in a 10-fold cross-validation using the genetic parameters estimated based on the sire-dam DHGLM and !BLUP option in ASReml. In total, four models were used in the 10-fold cross-validation, i.e. animal DHGLM with either ${\mathbf{A}}$ or ${\mathbf{H}}$ on stdWT and lnWT.

The 10-fold cross-validation was performed on standardized and transformed body weight data as follows:

1. Adjusted phenotypes for body weight ($y_{i}^{*}$) and its uniformity ($\psi_{i}^{*}$) were calculated as $y_{i}^{*} = \hat{a}_{i} + \hat{c}_{i} + \hat{e}_{i}$ and $\psi_{i}^{*} = \hat{a}_{{v_{i} }} + \hat{c}_{{v_{i} }} + \hat{e}_{{v_{i} }}$, using the solutions from the analysis with Eq. (2) on the full dataset.
2. In a modified dataset, approximately 10% of observed phenotypes ($y_{i}$) of animals from each family were masked (=10% of the full dataset). All phenotypes had an equal chance to be masked, but the animals that were masked in the previous fold were not masked again in the next fold.
3. The genetic analysis with Eq. (2) was run on the modified dataset using the ${\mathbf{A}}$ and ${\mathbf{H}}$ matrices and EBV for body weight and its uniformity were predicted for the masked animals.
4. For each fold, two measurements were computed:
  - The predictive ability of EBV was calculated as the Pearson correlation of adjusted phenotypes (step 1) with the corresponding EBV ($\hat{a}^{*}$) (step 3) for the masked animals that were genotyped, i.e., cor($y_{i}^{*}$, $\hat{a}_{i}^{*}$) for body weight and cor($\psi_{i}^{*}$, $\hat{a}_{{v_{i} }}^{*}$) for uniformity. Kendall and Spearman correlations were also calculated for uniformity because $\psi_{i}^{*}$ was exponentially rather than normally distributed.
5. To measure the degree of bias and accuracy of EBV or GEBV of the masked records, the mean square error prediction (MSEP) was calculated as $\frac{{\mathop \sum \nolimits_{i}^{n} \left( {\hat{a}_{i}^{*} - y_{i}^{*} } \right)^{2} }}{n}$ for body weight and $\frac{{\mathop \sum \nolimits_{i}^{n} \left( {\hat{a}_{{v_{i} }}^{*} - \psi_{i}^{*} } \right)^{2} }}{n}$ for uniformity of body weight, where $n$ is the number of masked records in each fold. The MSEP was scaled by the variance of the adjusted phenotypes of the corresponding trait.
6. Steps (1) to (4) were repeated for each of the 10 folds.

Finally, average Pearson, Kendall, and Spearman correlations, MSEP and their standard error (SE) over the 10 folds were calculated. A 95% confidence interval of the difference ($d$) in the predictive ability from different models with either ${\mathbf{A}}$ or ${\mathbf{H}}$ was constructed using $d \pm 1.96 \times {\text{SE}}_{d}$, where the ${\text{SE}}_{d} = \sqrt {\frac{{SD_{animal\,\,DHGLM}^{2} + SD_{{sire{\text{-} }dam \,\,DHGLM}}^{2} }}{number\,\,of\,\, folds}}$. When 0 was not within the 95% confidence interval, the predictive abilities of two models were considered statistically different (*P* < 0.05).

## Results

### Residual estimates

**Fig. 1:**
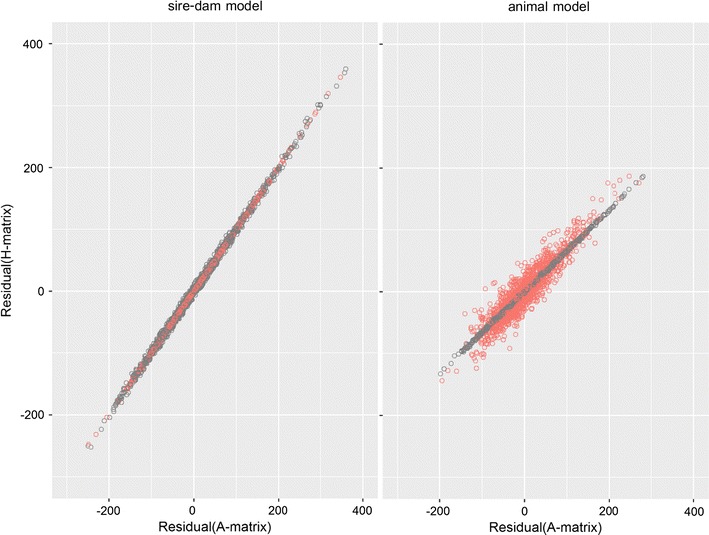
Scatter plot of residuals of body weight from the univariate analysis with ${\mathbf{A}}$ matrix (*x*-axis) or ${\mathbf{H}}$ matrix (*y*-axis). Two models were performed; sire-dam univariate model (*left*) and animal univariate model (*right*). *Red dots* are genotyped animals and *grey dots* are non-genotyped animals

Individual residuals estimated from using the ${\mathbf{A}}$ (BLUP) and ${\mathbf{H}}$ matrices (ssGBLUP) were plotted against each other to examine their relationship. As expected, the range of residual estimates from the animal models was lower than that from the sire-dam model since residual estimates from the sire-dam model included the entire Mendelian sampling term (Fig. 1).

For the sire-dam model, the use of ${\mathbf{H}}$ instead of ${\mathbf{A}}$ did not affect estimated residuals of genotyped animals since the regression coefficient of the estimated residuals using ${\mathbf{H}}$ on the estimated residuals using ${\mathbf{A}}$ and the Pearson correlation between the two were equal to 0.999, which was very similar to the regression coefficient of non-genotyped animals (0.998). The Pearson correlations between estimated residuals using ${\mathbf{H}}$ and ${\mathbf{A}}$ were the same as regression coefficients of estimated residuals using **A** on estimated residuals using **H** for genotyped animals (0.999) and non-genotyped animals (0.998).

In contrast, the use of ${\mathbf{H}}$ in the animal model affected residual estimates of genotyped animals since their distribution was much more scattered (Fig. 1). The slope of estimated residuals using **A** on estimated residuals using **H** was lower than 1 and slightly steeper for genotyped animals (regression coefficient = 0.7025) than for non-genotyped animals (regression coefficient = 0.6798). The Pearson correlations between estimated residuals using ${\mathbf{H}}$ and ${\mathbf{A}}$ were equal to 0.922 for genotyped animals and 0.966 for non-genotyped animals.

When using the sire-dam model, the difference in estimated residuals with ${\mathbf{H}}$ and ${\mathbf{A}}$ was small and ranged from −10.8 to 10.0. When using the animal model, this difference was larger and ranged from −95.3 to 104.5.

### Genetic parameters of body weight and its uniformity

For body weight, estimates of additive genetic variances from the sire-dam DHGLM with either ${\mathbf{A}}$ or ${\mathbf{H}}$ were similar (Table 2). Likewise, estimates of $h^{2}$ were similar with ${\mathbf{A}}$ and ${\mathbf{H}}$ for both traits: 0.266 and 0.296, respectively for stdWT and 0.325 and 0.346, respectively for lnWT.

**Table 2:** Estimates of variance components and genetic parameters of body weight and its uniformity based on the sire-dam double hierarchical generalized linear model when using pedigree (${\mathbf{A}}$) or combined pedigree and genomic relationships (${\mathbf{H}}$) and standard or log-transformed phenotypes

Trait/parameter	Standardization		Logarithm	
	${\mathbf{A}}$	${\mathbf{H}}$	${\mathbf{A}}$	${\mathbf{H}}$
Body weight				
$\sigma_{P}^{2}$	0.843	0.856	0.131	0.132
$\sigma_{a}^{2}$	0.216	0.243	0.043	0.046
$\sigma_{c}^{2}$	0.095	0.091	0.013	0.014
*h* ^2^	0.266^0.095^	0.296^0.102^	0.325^0.102^	0.346^0.107^
*c* ^2^	0.117^0.037^	0.111^0.037^	0.103^0.038^	0.103^0.038^
Uniformity of body weight				
$\sigma_{{a_{v} ,exp}}^{2}$	0.2303^0.1094^	0.2732^0.1211^	0.0896^0.0569^	0.0885^0.0598^
$\sigma_{{a_{v} }}^{2}$	0.0612	0.0677	0.0005	0.0005
$\sigma_{{c_{v} }}^{2}$	0.0360	0.0306	0.0000	0.0001
$GCV$	0.480^0.114^	0.523^0.116^	0.299^0.095^	0.298^0.100^
$h_{v}^{2}$	0.036^0.019^	0.038^0.020^	0.015^0.014^	0.014^0.013^
$c_{v}^{2}$	0.022	0.019	0.001	0.002

${\mathbf{A}}$ = pedigree based relationship matrix; ${\mathbf{H}}$ = combined genotyped and non-genotyped relationship matrix; $\sigma_{P}^{2}$ = phenotypic variance ($2\sigma_{u}^{2} + \sigma_{c}^{2} + \sigma_{e}^{2}$), where $\sigma_{e}^{2}$ is the residual variance for body weight; $\sigma_{a}^{2}$ and $\sigma_{{a_{v} }}^{2}$ = additive genetic variance for body weight and its uniformity, respectively; $\sigma_{c}^{2}$ = common environmental variance; *GCV* = coefficient of additive genetic variance for uniformity ($\sqrt {\sigma_{{a_{v} ,exp}}^{2} }$); *h* ^2^ = heritability for body weight; $c^{2}$ = common environmental effect due to full-sib tanks; $h_{v}^{2}$ = heritability for uniformity; $c_{v}^{2}$ = same as $c^{2}$ but for uniformity of body weight. Superscripts are SE of the estimates

When using ${\mathbf{A}}$, the estimate of $h_{v}^{2}$ was higher for uniformity of stdWT (0.036) than for uniformity of lnWT (0.015), while the use of ${\mathbf{H}}$ did not affect the magnitude of $h_{v}^{2}$ for uniformity. Standard errors of $h_{v}^{2}$ estimates were, however, high (Table 2).

Although the estimates of $h_{v}^{2}$ were low, estimates of $GCV$ were high for uniformity of stdWT (48.0% for ${\mathbf{A}}$ and 52.3% for ${\mathbf{H}}$), which indicates substantial genetic potential for response to selection. After accounting for scale effects, estimates of $GCV$ for uniformity of lnWT were reduced to 30% (for both ${\mathbf{A}}$ and ${\mathbf{H}}$), which supports the existence of genetic variation for uniformity beyond the scale effects.

Estimates of $c^{2}$ for stdWT and lnWT were moderate and similar for ${\mathbf{A}}$ (0.103 to 0.117) and ${\mathbf{H}}$ (0.103 to 0.111), which suggests that part of the phenotypic variation was explained by non-genetic effects that are common to full-sibs. Instead, the estimates of $c_{v}^{2}$ for uniformity of stdWT and lnWT were very low and ranged from 0.001 to 0.022 for ${\mathbf{A}}$ and 0.002 to 0.019 for ${\mathbf{H}}$ (Table 2).

The estimate of the genetic correlation between stdWT and its uniformity was close to 1, using either ${\mathbf{A}}$ (0.952) or ${\mathbf{H}}$ (0.951), which shows the high dependency between mean and variance of body weight. However, the estimate of the genetic correlation between lnWT and its uniformity was reduced to −0.093 with ${\mathbf{A}}$ and to 0.024 with ${\mathbf{H}}$, which suggests that after accounting for the scale effects, the mean and variance became independent.

### Cross-validation

**Fig. 2:**
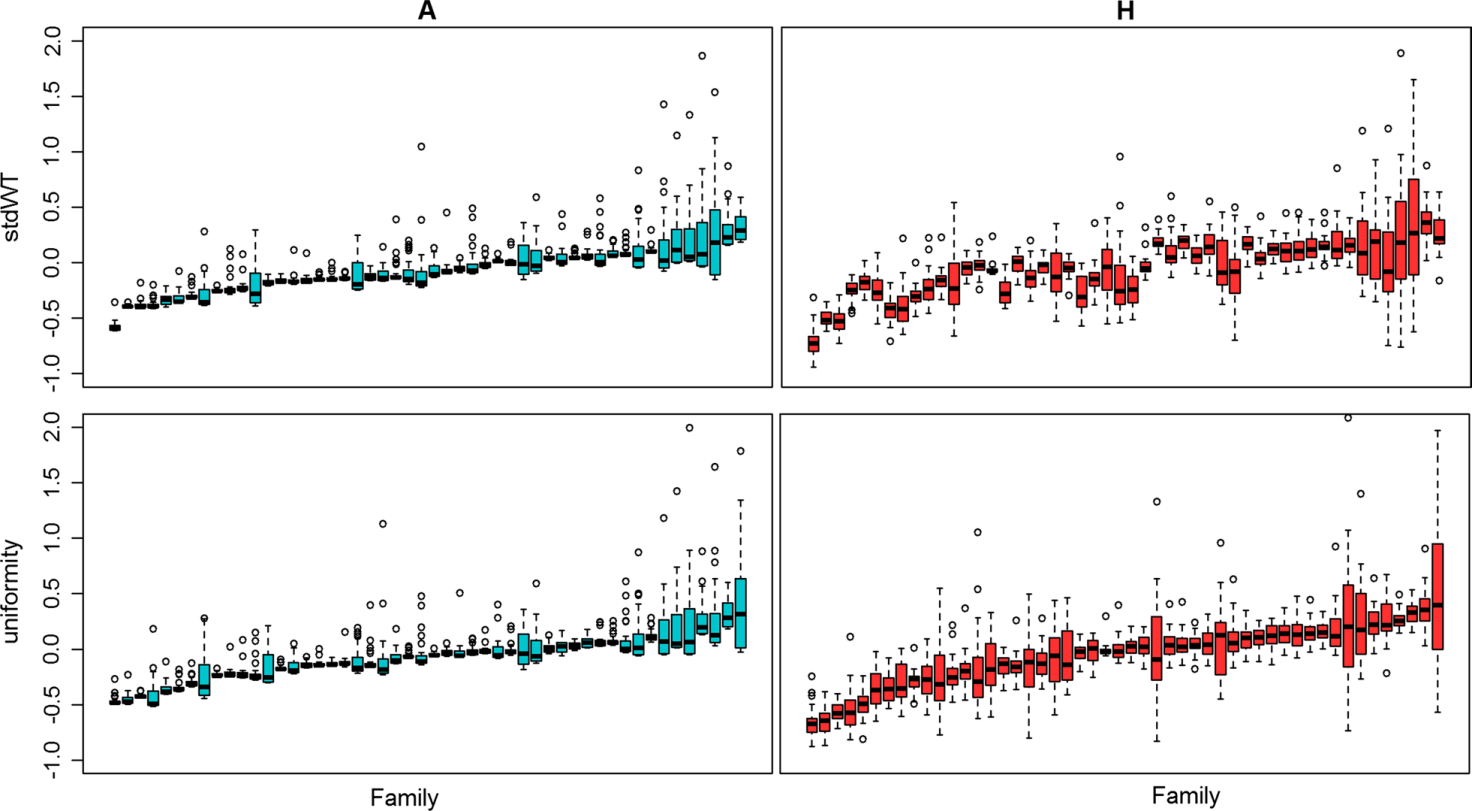
*Boxplots* of estimated breeding values for standardized body weight (stdWT) and its uniformity from genotyped animals by family. The breeding values were estimated using the animal double hierarchical generalized linear model. *Green boxplots* are estimated breeding values (EBV) using the ${\mathbf{A}}$ matrix and *red boxplots* are genomic estimated breeding values (GEBV) using the ${\mathbf{H}}$ matrix. The *x*-axis represents family identification

The use of ${\mathbf{H}}$ instead of ${\mathbf{A}}$ with the animal DHGLM resulted in more variation of the within-family GEBV for stdWT and its uniformity, compared to within-family EBV (Fig. 2; Additional file 1: Figure S1), for sire-dam DHGLM).

The average correlation of adjusted phenotypes with predicted breeding values for stdWT and its uniformity was significantly higher with ${\mathbf{H}}$ (stdWT = 0.443; uniformity = 0.217 to 0.317) than with ${\mathbf{A}}$ (stdWT = 0.372; uniformity = 0.128 to 0.192). However, after accounting for scale effects using log-transformation, the average Pearson, Kendall and Spearman correlations of adjusted phenotypes with predicted breeding values for lnWT and their uniformity were only slightly higher with ${\mathbf{H}}$ than with ${\mathbf{A}}$, and not significantly different from each other (*P* > 0.05).

The average MSEP for uniformity from the animal DHGLM (0.608 to 0.944) were lower than those from the sire-dam DHGLM (0.973 to 1.112), suggesting that the use of an animal DHGLM increases the accuracy and may reduce bias in predicting breeding values for uniformity (Table 3; Additional file 2: Table S1). However, the average MSEP for uniformity of stdWT and lnWT obtained with ${\mathbf{H}}$ (0.608 to 0.944) were not notably different from those obtained with ${\mathbf{A}}$ (0.625 to 0.936).

**Table 3:** Average Pearson, Kendall and Spearman correlations and mean square error prediction from a 10-fold cross-validation based on the sire-dam double hierarchical generalized linear model^a^ when using pedigree (${\mathbf{A}}$) or combined pedigree and genomic relationships (${\mathbf{H}}$) and standard or log-transformed phenotypes

Transformation	Relationship	Body weight		Uniformity of body weight			
		Pearson	MSEP	Pearson	Kendall	Spearman	MSEP
Standardized	${\mathbf{A}}$ matrix	0.372^0.013^	0.724^0.021^	0.192^0.033^	0.128^0.021^	0.178^0.030^	0.625^0.086^
	${\mathbf{H}}$ matrix	0.443^0.017^	0.682^0.021^	0.271^0.018^	0.217^0.017^	0.317^0.025^	0.608^0.082^
Logarithm	${\mathbf{A}}$ matrix	0.396^0.019^	0.823^0.029^	0.378^0.032^	0.182^0.016^	0.263^0.023^	0.936^0.085^
	${\mathbf{H}}$ matrix	0.440^0.016^	0.813^0.028^	0.383^0.026^	0.203^0.014^	0.294^0.020^	0.944^0.085^

^a^The variance components from the sire-dam double hierarchical generalized linear model were converted to the animal double hierarchical generalized linear model and were used in the 10-fold cross-validation. Relationship = relationship matrix, where ${\mathbf{A}}$ refers to pedigree-based relationship matrix and ${\mathbf{H}}$ refers to combined genotyped and non-genotyped relationship matrix. The predictability was calculated as the Pearson, Kendall and Spearman correlations between marked phenotype and predicted breeding value. MSEP was scaled by the phenotypic variance of corresponding traits

The predictive ability of EBV of uniformity was sensitive to the type of correlation used, i.e. Pearson, Kendall and Spearman (Table 3). Spearman correlations were 39.1 to 49.0% higher than Kendall correlations. Predictive abilities of EBV and GEBV for uniformity of lnWT differed more from each other based on Kendall and Spearman correlations, albeit not significant at *P* < 0.05, than based on Pearson correlations. However, the SE of Kendall correlations were approximately 50% lower than the SE of Pearson and Spearman correlations, suggesting that Kendall correlations provide a more reliable estimate of predictive ability than Pearson and Spearman correlations.

## Discussion

To the best of our knowledge, this is the first study that compares the use of the numerator relationship (${\mathbf{A}}$) and a combined genomics and numerator relationship (${\mathbf{H}}$) matrix for estimating genetic parameters and predicting breeding values for body weight and its uniformity. The use of the animal DHGLM with ${\mathbf{H}}$ significantly improved the predictive ability of GEBV for uniformity of body weight (stdWT) but not for scale-adjusted uniformity.

### Genetic parameters

The estimate of heritability for uniformity of stdWT from sire-dam DHGLM with ${\mathbf{A}}$ was low ($h_{v}^{2}$ = 0.036) but higher than estimates of $h_{v}^{2}$ obtained in previous studies on rainbow trout [4, 8] and Nile tilapia [15, 16] ($\bar{h}_{v}^{2}$ = 0.016: min = 0.010: max = 0.024). However, after accounting for scale effects by logarithm transformations, the estimate of $h_{v}^{2}$ decreased to 0.014 to 0.015, which is in line with the previous reports that also used transformations [4, 8, 14–16].

Estimates of $h_{v}^{2}$ for stdWT and lnWT using the sire-dam DHGLM with ${\mathbf{H}}$ did not differ from those with ${\mathbf{A}}$, which is in line with estimates of $h_{v}^{2}$ for uniformity of piglet birth weight obtained using either ${\mathbf{A}}$ or only the genomic relationship matrix (${\mathbf{G}}$) [42], while lower estimates were reported for environmental variance of somatic cell score in dairy cattle when using ${\mathbf{G}}$ compared to ${\mathbf{A}}$ [43]. The similarity of the estimates of $h_{v}^{2}$ obtained by using ${\mathbf{A}}$ or ${\mathbf{H}}$ in this study can be explained by the very similar estimated residuals (proxy of uniformity) between non-genotyped and genotyped animals when using the sire-dam model with ${\mathbf{A}}$ and ${\mathbf{H}}$. The sire-dam model only exploits relationships between sires and dams and does not exploit the full potential of the genotype-based relationships between animals, and especially between full-sibs. In contrast, residuals of genotyped animals estimated by using the animal model were more differentiated when either ${\mathbf{A}}$ or ${\mathbf{H}}$ was used, and likely more accurate than estimates of residuals for non-genotyped animals. However, in a DHGLM analysis, the sire-dam model provides less biased (co)variance components than the animal model [14], likely because of the dependence between estimates of the breeding value and residual of an individual, which are obtained from the same phenotype of body weight. The use of genomic relationships combined with numerator relationships is expected to reduce the dependency between EBV and estimated residuals because the EBV are more accurate. Therefore, we performed the animal DHGLM with ${\mathbf{H}}$ but the model did not converge when the variance components were estimated, which may be due to (1) the dependency between EBV and estimated residuals for body weight remaining high, or (2) the difficulty to disentangle genetic effects from the common environmental effects for uniformity of body weight.

The standard errors of $h_{v}^{2}$ estimates were high, which may be due to the large variation in family size (4 to 54). According to Hill and Mulder [30], large family sizes or repeated measurements are recommended for estimating the genetic heteroscedasticity of traits. The optimal full-sib family size is 39 with a $GCV$ of 39% and an $h^{2}$ of 0.36 [18]. The use of ${\mathbf{H}}$ did not affect the standard errors of the $h_{v}^{2}$ estimates, which does not agree with previous studies, for example Veerkamp et al. [44] reported lower standard errors of $h^{2}$ estimates for dry matter intake, milk yield, and body weight of heifers when using genomic relationships with an animal model. One possible explanation could be that the benefit of using the genomic relationship matrix may be limited when the sire-dam DHGLM is applied, since the variance of genomic relationships between sires (0.02) was very similar to the variance of numerator relationships between sires (0.01). In contrast, the variance in genomic relationships between animals (0.01) was much larger than the variance of numerator relationships between animals (0.004). Hence, the SE of $h_{v}^{2}$ estimates may be lower when using an animal DHGLM with a genomic relationship matrix, compared to the numerator relationship matrix. Nevertheless, in our study, it was not possible to investigate this phenomenon since the log-likelihood did not converge for the animal DHGLM with ${\mathbf{H}}$.

The $GCV$ of uniformity for stdWT was substantial (48.0%), which indicates high potential for response to selection. This result is in the upper range of previous findings in fish species (17.4 to 64.0%) [4, 8, 14–16, 19] and in terrestrial animals (10.0 to 58.0%) [6, 9–13, 42]. After accounting for scale effects by logarithm transformations, the $GCV$ for uniformity was reduced but still substantial (29.5 to 29.9%), which was also reported in previous studies on Atlantic salmon [14], rainbow trout [8], rabbit, and pig [45]. Thus, scale effects affect estimates of genetic parameters for uniformity of body weight considerably, but there is genetic variation for uniformity beyond the scale effect.

Genomic information slightly increased the $GCV$ of uniformity of stdWT (from 48.0 to 52.3%). In contrast, genomic information did not influence the $GCV$ for uniformity of lnWT (29.8%). Since estimates of genetic parameters obtained with ${\mathbf{A}}$ and ${\mathbf{H}}$ were similar, the $GCV$ for body weight remained similar, which is in agreement with the previous comparison between ${\mathbf{A}}$ ($GCV$ = 11.0 to 12.0%) and ${\mathbf{G}}$ ($GCV$ = 10.0 to 11.0%) for uniformity of birth weight of piglets using a dam model [42].

### Genetic and genomic predictions

In this study, we used the Pearson correlation of EBV and GEBV with adjusted phenotype as the measure of predictive ability. The use of ${\mathbf{H}}$ instead of ${\mathbf{A}}$ in the animal DHGLM significantly improved the ability to predict breeding values for stdWT (19%) and its uniformity (41.1 to 78.1%). Furthermore, the use of the animal DHGLM instead of the sire-dam DHGLM significantly increased the predictive ability of EBV and GEBV for uniformity (see Additional file 2: Table S1), as expected.

Our findings indicate that ssGBLUP with an animal DHGLM can increase the accuracy of EBV for uniformity substantially compared to pedigree-based BLUP. However, after accounting for scaling effects by using log transformations, the use of ${\mathbf{H}}$ compared to ${\mathbf{A}}$ only slightly improved the correlation (1.6 to 13.9%) and MSEP between GEBV and adjusted phenotypes, and the improvement was not significant. There are two main reasons why these results differed between uniformity of stdWT and lnWT. First, log-transformation substantially reduced the genetic correlation of stdWT with its uniformity. As a result, any increases in predictive ability of GEBV of lnWT when using ${\mathbf{H}}$ instead of ${\mathbf{A}}$ (15.7%) did not positively influence the predictive ability of GEBV of its uniformity. Second, the lower additive genetic variance and $h_{v}^{2}$ for uniformity after accounting for scale effects reduces the accuracy of EBV for uniformity. Consequently, MSEP increased from 0.63 (stdWT) to 0.94 (lnWT) with ${\mathbf{A}}$ and from 0.61 (stdWT) to 0.94 (lnWT) with ${\mathbf{H}}$ in the animal DHGLM.

The accuracy of genomic selection is expected to increase when the number of genotyped animals in the reference population increases for any trait [46] but in particular for lowly heritable traits, such as uniformity as shown by Sell-Kubiak et al. [42] and somatic cell score by Mulder et al. [43]. In this study, uniformity of lnWT had an even lower $h_{v}^{2}$ than uniformity of stdWT. The number of genotyped animals in the reference population used for cross-validation was on average equal to 1274, which may have limited the benefit of using genomic information in ssGBLUP. A future empirical study should investigate the effect of the number of genotyped animals in the reference population on the ability to predict breeding values for uniformity to validate our findings and conclusions.

### Pearson or rank correlations?

Squared residuals or adjusted phenotype for uniformity ($\psi_{i}^{*} )$ are exponentially rather than normally distributed, which may not justify quantifying predictive ability using a Pearson correlation. Thus, we also calculated distribution-free rank correlations (Kendall and Spearman) and indeed found estimation of the predictive ability for uniformity to be sensitive to the type of correlation used.

Although not significantly different, Kendall and Spearman correlations explained differences in predictive ability of EBV and GEBV for uniformity of lnWT slightly better than the Pearson correlation. Hence, the conclusion that the benefit of using genomic relationship for computing EBV for uniformity of logarithm transformations is limited remained the same when using Kendall and Spearman correlations. Colwel and Gillett [47] showed that, in general, estimates of Kendall correlations are similar to estimates of Spearman correlations, but in some cases, the magnitude of Spearman correlations can be 50% greater than the magnitude of Kendall correlations [47]. This is in line with our findings since Spearman correlations were 42.2 to 49.0% and 39.1 to 46.1% greater than Kendall correlations for the sire-dam DHGLM (see Additional file 2: Table S1) and the animal DHGLM, respectively. Nevertheless, the SE of Kendall correlations were notably lower by approximately 50% than the SE of Pearson and Spearman correlations, which indicates that the Kendall correlation may be a more reliable estimate of predictive ability than the Pearson and Spearman correlations. Hence, it is recommended to use Kendall instead of Pearson correlations when studying predictive ability for uniformity.

### Selection for uniformity

For fish breeding, major goals are to increase mean body weight and reduce variability (more uniformity) of body weight. Nevertheless, definitions of uniformity of stdWT and lnWT are not the same. From a biological point of view, genetic variation for environmental canalization can be quantified after the scale effect is accounted for. However, from an animal breeding point of view, uniformity on the observed scale explains the actual range of fish sizes that are processed by aquaculture industries.

Selection for body weight and uniformity may be challenging because the genetic correlation between body weight and its variability is high and positive, and sometimes approaches 1. A general observation is that the genetic correlation between log-transformed body weight and its variability is zero or even negative, allowing selection to simultaneously increase transformed body weight and reduce variability. Therefore log-transformed body weight and its variability could be included in a selection index. This would require knowledge of the genetic correlation between variability of stdWT and variability of lnWT, which is not available. Hence, we used the sire-EBV and sire-GEBV, obtained from BLUP and ssGBLUP with the animal DHGLM, to calculate the Pearson correlations between EBV for the trait and its variability. Pearson correlations between EBV for variability of stdWT and EBV for variability of lnWT were positively moderate with BLUP (0.48) and ssGBLUP (0.68). The Pearson correlation between EBV for stdWT and EBV for its variability was close to 1 with either BLUP (0.96) or ssGBLUP (0.97), while the Pearson correlation between EBV of lnWT and its variability was −0.24 for BLUP and -0.005 for ssGBLUP. Not surprisingly, the Pearson correlation between EBV for lnWT and EBV for stdWT was highly positive (0.82). These Pearson correlations suggest that variability of lnWT should be included in the selection index because the GEBV for variability of lnWT are positively correlated with GEBV for variability of stdWT, which indicates that selection against variability of lnWT will indirectly reduce variability on the observed scale. Furthermore, GEBV for variability of lnWT are not correlated to GEBV for lnWT and thus selection against variability of lnWT will not indirectly reduce lnWT.

To elucidate responses to selection for body weight and its variability, we performed truncation selection on sires based on their GEBV from the animal DHGLM with ssGBLUP. The breeding goal could include body weight and variability on the observed scale and their economic values ($v$): $v_{1} {\text{BV}}_{\text{stdWT}} - v_{2} {\text{BV}}_{{{\text{variability}}_{\text{stdWT}} }}$, while, based on the Pearson correlations discussed above, the selection index (${\text{I}}$) could include lnWT and its variability with their relative weighting factors ($b$):

$${\text{I}} = b_{1} {\text{GEBV}}_{\text{lnWT}} - b_{2} {\text{GEBV}}_{{{\text{variability}}_{\text{lnWT}} }} .$$

Selecting the 10% best sires on an index with $b_{1}$ of 0.3, i.e., ${\text{I}} = 0.3 * {\text{GEBV}}_{\text{lnWT}} - 0.7 * {\text{GEBV}}_{{{\text{variability}}_{\text{lnWT}} }}$ provides almost no genetic gain in variability of stdWT (−0.001) but positive genetic gain in stdWT (3.62% of mean body weight in g). In contrast, a selection index, based on breeding goal traits:${\text{I}} = 0.52 * {\text{GEBV}}_{\text{stdWT}} - 0.48 * {\text{GEBV}}_{{{\text{variability}}_{\text{stdWT}} }}$, provides zero genetic gains for both stdWT and its variability, showing no possibility to achieve genetic gain on body weight while maintaining stable phenotypic variability. Nevertheless, the genetic gain in stdWT was much greater (17.32% of mean body weight in g) when variability was not included in the selection index. Therefore, although it is possible to increase body weight while keeping variability constant, there is a trade-off in genetic gain for body weight when selecting for reduced variability.

## Conclusions

The use of the animal DHGLM instead of the sire-dam DHGLM substantially increased the predictive ability for breeding values of uniformity, because the animal DHGLM fully exploits the relationships between full- and half-sibs. When using the animal DHGLM, the use of a combined numerator and genomic relationship matrix significantly increased the predictive ability for breeding values of uniformity of body weight, but only a slight and non-significant increase was observed after accounting for the scale effects by using transformed body weights. The small increase in predictive ability with transformed body weights may be due to lower heritability for uniformity of transformed body weight, a lower genetic correlation between transformed body weights and their uniformities, and/or a small number of genotyped animals in the reference population. The use of a Kendall correlation provided the lowest SE of predictive ability for uniformity and provided a more accurate estimate of predictive ability for uniformity over Pearson and Spearman correlations. In conclusion, the use of ssGBLUP increases the accuracy of breeding values for uniformity of harvest weight, which is expected to increase response to selection in uniformity.

## Additional files

**Additional file 1: Figure S1.** Estimated breeding values for standardized body weight and its uniformity based on sire-dam DHGLM. The data provided represent the boxplot of estimated (genomic) breeding values of genotyped animals based on sire-dam DHGLM when using pedigree (**A**) or combined pedigree and genomic relationships (**H**).

**Additional file 2: Table S1.** Cross-validation based on sire-dam DHGLM. The data provided represent the results from 10-fold cross-validations based on sire-dam DHGLM.
