# Supplementary material for: Estimation of breeding values for uniformity of growth in Atlantic salmon (Salmo salar) using pedigree relationships or single-step genomic evaluation
Source: Genet Sel Evol. 2017 Mar 7;49:33. doi: 10.1186/s12711-017-0308-3 (PMC5439168; doi:10.1186/s12711-017-0308-3)
Supplement: Supplementary file 1 — Additional file 1: Figure S1. Estimated breeding values for standardized body weight and its uniformity based on sire-dam DHGLM. The data provided represent the boxplot of estimated (genomic) breeding values of genotyped animals based on sire-dam DHGLM when using pedigree (A) or combined pedigree and genomic relationships (H). [file 12711_2017_308_MOESM1_ESM.pdf]

Additional file 1

Estimated breeding values for standardized body weight (stdWT) and its uniformity from genotype animals based on sire-dam DHGLM

The use of **H** with sire-dam DHGLM resulted in higher variation of within-family GEBVs for stdWT and its uniformity, compared to within family EBVs (Figure. S1). However, the animal DHGLM resulted in considerably higher variation of within-family EBVs and GEBVs for stdWT and its uniformity, compared to sire-dam DHGLM (see Figure. 2).

Figure S1 Boxplot of estimated breeding values of standardized body weight (stdWT) and its uniformity from genotyped animals by family. The breeding values were estimated

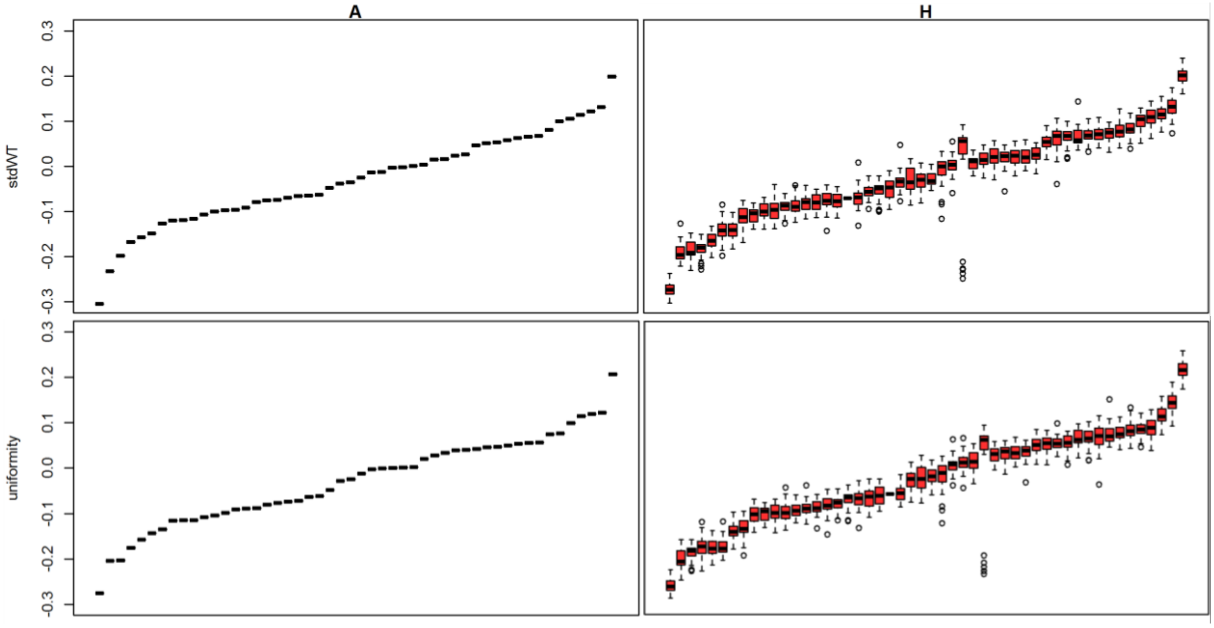

using sire-dam double hierarchical generalized linear model. The green boxplots are estimated breeding values (EBVs) using **A** matrix while the red boxplots are genomic estimated breeding values (GEBVs) using **H** matrix. The x-axis is the family identification.
