# Supplementary material for: Estimation of breeding values for uniformity of growth in Atlantic salmon (Salmo salar) using pedigree relationships or single-step genomic evaluation
Source: Genet Sel Evol. 2017 Mar 7;49:33. doi: 10.1186/s12711-017-0308-3 (PMC5439168; doi:10.1186/s12711-017-0308-3)
Supplement: Supplementary file 2 — Additional file 2: Table S1. Cross-validation based on sire-dam DHGLM. The data provided represent the results from 10-fold cross-validations based on sire-dam DHGLM. [file 12711_2017_308_MOESM2_ESM.pdf]

Additional file 2

Cross validation based on sire-dam DHGLM

Due to different contents of squared residual when using either sire-dam or animal model, the predictive ability for estimated breeding values using sire-dam DHGLM with either **A** or **H** were investigated using 10-fold cross validation, described in **Genetic evaluation and cross validation** section.

The average Pearson correlations between adjusted phenotype and EBVs for stdWT and lnWT with **H** were slightly higher (0.285 to 0.308) than that with **A** (0.264 to 0.279) but not significantly different ($P > 0.05$). Likewise, the respective average Pearson correlations for uniformity of stdWT with **H** (0.105) and with **A** (0.110) were not significantly different ($P > 0.05$). Hence, the use of **H** did not improve the predictive ability of estimated breeding values of stdWT and it uniformity when using a sire-dam DHGLM. After accounting for the scale effects, the average Pearson correlations for uniformity became considerably lower. Although average Pearson correlations for uniformity of lnWT with **H** were slightly higher than that with **A**, they were not significantly different ($P > 0.05$). The average MSEPs for body weight estimated from sire-dam DHGLM (Table S1) with **A** (0.786 to 0.897) and **H** (0.789 to 0.904) were very similar, indicating that **H** did not significantly improve the accuracy of EBVs for body weight when using sire-dam DHGLM. Likewise, the average MSEPs for uniformity of body weight when using **A** (0.973 to 1.109) or **H** (0.975 to 1.112) were in a very similar range.

Table S1 Average Pearson, Kendall and Spearman correlations and mean square error prediction (MSEP) from 10-fold cross validation using sire-dam double hierarchical generalized linear model

Transformation	Relationship	Body weight		Uniformity of body weight			
		Pearson	MSEP	Pearson	Kendall	Spearman	MSEP
Standardized	**A** matrix	$0.264^{0.014}$	$0.786^{0.024}$	$0.110^{0.019}$	$0.192^{0.011}$	$0.273^{0.016}$	$1.109^{0.150}$
	**H** matrix	$0.285^{0.013}$	$0.789^{0.024}$	$0.105^{0.013}$	$0.158^{0.010}$	$0.228^{0.014}$	$1.112^{0.150}$
Logarithm	**A** matrix	$0.279^{0.020}$	$0.897^{0.033}$	$0.057^{0.030}$	$0.075^{0.016}$	$0.110^{0.025}$	$0.973^{0.105}$
	**H** matrix	$0.308^{0.018}$	$0.904^{0.033}$	$0.072^{0.025}$	$0.096^{0.011}$	$0.143^{0.017}$	$0.975^{0.105}$

Relationship = relationship matrix, where **A** refers to pedigree relationship matrix and **H** refers to combined genotyped and non-genotyped relationship matrix. The predictability was calculated as the Pearson, Kendall and Spearman correlations between marked phenotype and predicted breeding value. MSEP was scaled by the phenotypic variance of corresponding traits.

The use of animal DHGLM instead of sire-dam DHGLM significantly increased the predictive ability of EBV and GEBV for uniformity by at least 73.9% for **A** and 156.8% for **H,** and noticeably reduced MSEP by 3.2% to 45.3%. Hence, our results suggest that animal DHGLM with BLUP run using (co)variance components from sire-dam DHGLM provides considerable improvement of ability in predicting breeding values for uniformity of body weight compared to sire-dam DHGLM. There are three possible reasons for this phenomenon.

First, the BLUP run with animal DHGLM exploits the full potential of realized relationship matrix, especially for full-sibs, which yields more differentiated individual estimated residuals, and thus more accurate estimation of both phenotypes and breeding values of uniformity. Second, the animal DHGLM includes own performance as the information source, increasing the precision of the residuals. Third, unlike sire-dam DHGLM, animal DHGLM results in the individual estimated residuals without Mendelian sampling term. Thus, the use of animal DHGLM with BLUP run results in different trait definition for uniformity compared to sire-dam DHGLM.
